# The specific and rapid labeling of cell surface proteins with recombinant FKBP-fused fluorescent proteins

**DOI:** 10.1007/s13238-014-0090-8

**Published:** 2014-08-12

**Authors:** Xi Zhang, Yongqiang Deng, Hao Chang, Chen Ji, Mingshu Zhang, Jianxin Peng, Tao Xu, Pingyong Xu

**Affiliations:** 1 Institute of Entomology, School of Life Sciences, Central China Normal University, Wuhan, 430079 China; 2 National Key Laboratory of Biomacromolecules, Institute of Biophysics, Chinese Academy of Sciences, Beijing, 100101 China; 3 School of Life Sciences, University of Science and Technology of China, Hefei, 230026 China; 4 Laboratory of Interdisciplinary Research, Institute of Biophysics, Chinese Academy of Sciences, Beijing, 100101 China

**Dear Editor,**

The presence and abundance of proteins in the plasma membrane (PM) is regulated by exocytosis and endocytosis. Exocytosis is required for the maintenance of cellular homeostasis following stimulation, whereas endocytosis is important for the reuse and degradation of PM and receptor proteins. Through the specific labeling of proteins on the PM, the process of endocytosis can be tracked and monitored in living cells. Several genetic tags and site-specific labeling approaches that involve the coupling of small organic molecules to tag-fused proteins through either self-labeling or enzymatic ligation have been developed for this purpose (Chen et al., 2005; George et al., 2004; Hoffmann et al., 2010; Keppler et al., 2003; Los et al., 2008; Uttamapinant et al., 2010). However, these methods require a long time for an efficient labeling.

Techniques with slow labeling rates are not suitable for live cell imaging. This is especially true for tracking proteins that are rapidly cycled to and from the PM in neurons because the integrity and function of these proteins require extremely fast membrane trafficking. A typical neuron in the human brain fires action potentials at 10 Hz, causing the fusion of several hundred synaptic vesicles every second (Sudhof, 2004). Accordingly, the rapid endocytosis of extensive transmembrane proteins following their exocytosis is required for the local regeneration of synaptic vesicles at the synaptic terminals. At present, fast endocytosis cannot be effectively traced using the available chemical probe-based labeling methods. Sun et al. have recently reported a fast-labeling variant of the SNAP-tag, termed SNAP_f_, which displays up to a tenfold increase in its reactivity towards benzylguanine substrates (Sun et al., 2011). The time required for 50% labeling of SNAP_f_ (t_1/2_) is 11 to 34 s for different SNAP-surface BG dyes (Sun et al., 2011). However, the widespread use of SNAP_f_ remains limited by the background fluorescence from unreacted or non-specifically bound substrates. Moreover, a thorough wash step is required to reduce the fluorescence signals of unreacted dyes for the vast majority of chemical labeling processes, including SNAP_f_-tag labeling; this requirement restricts certain labeling applications, such as the real-time imaging of the endocytosis of membrane proteins. To overcome this difficulty, Sun et al. developed fluorogenic SNAP-tag probes with low background fluorescence that become highly fluorescent only upon reaction with their target proteins (Sun et al., 2011). However, the incorporation of intramolecular quenchers greatly reduces the reactivity of the fluorogenic substrates for both SNAP-tag and SNAP_f_-tag (Sun et al., 2011), resulting in a slow labeling rate, with t_1/2_ ranging from 3 to 18 min for SNAP_f_-tag (Sun et al., 2011). Thus, a strong need still remains for an efficient labeling method that can combine both a fast labeling rate (within tens of seconds) and high specificity with real-time detection and high-contrast imaging.

To achieve this objective, we developed a strategy to label cell surface proteins very quickly and with high specificity in living cells. The technique uses purified FKBP-fused fluorescent proteins (FPs) that binds to AP21967, a rapamycin analogue that has a high binding affinity to the FRB mutant (T2098L) but not to native FRB (Banaszynski et al., 2006). We termed this approach LAPREP, which stands for labeling PM proteins with recombinant FKBP-tagged fluorescent proteins. The labeling scheme is shown in Fig. 1A.

**Figure 1:**
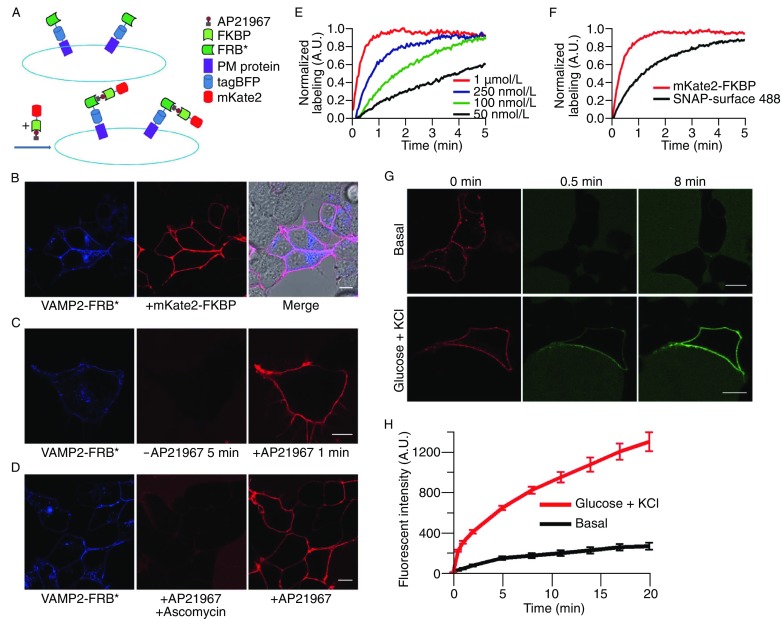
Labeling PM proteins with FKBP-tagged fluorescent proteins. (A) Labeling scheme. Interaction between FKBP-AP21967 with FRB (T2098L) domain on the PM results in specific binding of fluorescence to cell surface proteins. FRB* means the mutation FRB T2098L. (B) Labeling of HEK293 cells transiently expressing VAMP2-TagBFP-FRB (T2098L). (C) HEK293 cells transfected with VAMP2-TagBFP-FRB (T2098L) were first incubated with mKate2-FKBP for 5 min without AP21967, then incubated with mKate2-FKBP with AP21967 for 1 min when images were acquired. (D) mKate2-FKBP-AP21967 with ascomycin was first added to the transfected HEK293 cells surface, then incubated with mKate2-FKBP with AP21967. (E) Different concentrations of AP21967 were mixed with the same concentration of mKate2-FKBP (1 µmol/L). Real-time imaging was applied to monitor the intensity on the PM. (F) mKate2-FRB mixed with 1 µmol/L AP21967 labeling was compared with SNAP-tag (1 µmol/L) labeling. (G) INS-1 cells were transfected with VAMP2-TagBFP-FRB (T2098L). The PM pre-existing VAMP2 were first labeled with mKate2-FKBP-AP21967 for 5 min, and GFP-FKBP-AP21967 was then added to chase the new arrival of VAMP2. The fluorescent intensity on the basal cell surface is very weak at 30 s, proving the pre-existing VAMP2 had been completely blocking. (H) Kinetics of VAMP2 inserting into PM during insulin secretion

We chose VAMP2, an important V-SNARE protein for vesicle fusion, as a target protein and fused the FRB (T2098L) domain to the C-terminus of VAMP2. FRB (T2098L) faces the extracellular region when it is at the PM, allowing contact between FKBP and FRB. To discriminate between VAMP2-expressing and VAMP2-non-expressing cells, we fused a blue FP, TagBFP, to the region immediately preceding FRB. The purified chimeric protein mKate2-FKBP, which we generated by fusing FKBP with mKate2, was mixed with AP21967 and added to HEK293 cells expressing VAMP2-TagBFP-FRB (T2098L). We found that mKate2-FKBP-AP21967 effectively labeled VAMP2-FRB (T2098L) in cells expressing the target protein marked by TagBFP but not in cells without blue fluorescence, suggesting the high specificity of the labeling (Fig. 1B). The specific binding is dependent on AP21967, given that without AP21967, no VAMP2-TagRFP-FRB was labeled on the PM (Fig. 1C). Moreover, the binding of mKate2-FKBP to VAMP2-TagRFP-FRB can be partially inhibited by the co-incubation of the cells with ascomycin, which is a competitive binding partner for FKBP (Fig. 1D).

We then checked the labeling efficiency of VAMP2 by LAPREP. As shown in Fig. 1C, the defined filopodial structure can be labeled by LAPREP; this result likely indicates the high efficiency of the labeling. To further support this hypothesis, we analyzed the binding kinetics of mKate2-FKBP-AP21967 to VAMP2-FRB (T2098L) in living cells. In particular, we changed the concentration of AP21967 from 50 nmol/L to 1 μmol/L while maintaining a constant mKate2-FKBP concentration of 1 μmol/L. Because the interaction between AP21967 and the FKBP domain is very strong, and the concentration of FKBP was much higher than that of AP21967, the concentrations of AP21967 in this experiment represent the effective amount of the mKate2-FKBP-AP21967 complex that is able to bind FRB (T2098L).

As shown in Fig. 1E, higher concentrations of FKBP-AP21967 complex lead to an increasing labeling efficiency and a more rapid rate of mKate2-FKBP binding to VAMP2-FRB (T2098L) on the PM. At lower concentrations of the FKBP-AP21967 complex (50 nmol/L and 100 nmol/L, Fig. 1E), several minutes are required for the labeling of most of the target cell surface proteins, whereas at the higher concentration of 1 μmol/L, the majority of the VAMP2-TagRFP-FRB (90%) was labeled within 1 min (Fig. 1E). Most importantly, extremely low background fluorescence was observed at all concentrations of mKate2-FKBP-AP21967, without apparent differences for distinct AP21967 concentrations. We then compared the labeling dynamics between the FKBP-AP21967 and SNAP-tag approaches in living cells. As shown in Fig. 1F, the t_1/2_ (time required to label half of the cell surface proteins) was much smaller for the FKBP-AP21967 method (13 s) than for the SNAP-Surface 488 method (72 s), which is consistent with a previously published report (Sun et al., 2011). These results indicate that labeling with the FKBP-AP21967 method is much more rapid than SNAP-tag labeling and that the fluorescence background of the FKBP-AP21967 method is extremely low.

Next, we checked the dynamic labeling of VAMP2 on the PM by LAPREP. VAMP2 is a SNARE protein that is located on the insulin-containing vesicles that are responsible for insulin secretion in endocrine pancreatic beta cells (Regazzi et al., 1995). Under resting conditions, VAMP2 cycles slowly between vesicles and the PM (Grote and Kelly, 1996). Upon stimulation with high glucose or potassium, insulin granules fuse with the PM and rapidly secrete insulin, causing VAMP2 to insert into the PM (Eliasson et al., 2008). To observe VAMP2 dynamics under resting and stimulated conditions, we performed sequential pulse-chase labeling of VAMP-FRB (T2098L) with mKate2-FKBP-AP21967 and EGFP-FKBP-AP21967. We demonstrated that a large amount of VAMP2 resided on the PM under resting conditions in INS-1 cells, as indicated by the red fluorescence of mKate2, while a small amount of VAMP2 was constitutively inserted into the PM and selectively labeled with EGFP-FKBP-AP21967 within 10 min (Fig. 1G and 1H). Upon stimulation with high glucose and potassium, significantly greater quantities of VAMP were inserted into the PM and could be labeled by the pulse-chased EGFP-FKBP-AP21967 (Fig. 1H). Using this method, the dynamic amounts of VAMP2 could be monitored (Fig. 1H).

Last, we studied the retrograde trafficking mechanism of VAMP2 in the endocrine INS-1 cell line. As mentioned above, there are two fractions of VAMP2 in the PM: an already-present fraction that reflects the basal/constitutive insulin secretion and a newly inserted fraction that reflects the stimulated insulin secretion. It is unclear whether these two fractions are distinctly endocytosised in INS-1 cells. We sequentially labeled these two fractions by pulse chase with mKate2- and EGFP-fused FKBP-AP21967 and monitored the colocalization of the two tagged proteins after 15 min. As shown in Fig. S1, sequentially labeled VAMP2 was retrograde trafficked back into the cells, where the labels colocalized very well, suggesting that the two fractions underwent the same retrograde trafficking process.

It has been reported that VAMP2 enters cells via clathrin-dependent endocytosis (Dittman and Ryan, 2009; Harel et al., 2008; Miller et al., 2011). To investigate whether the LAPREP method could be used to monitor this process without affecting the recycling of VAMP2, we examined the colocalization of endocytic VAMP2 and transferrin, which is a general marker for clathrin-dependent endocytosis. The transfected cells were incubated with mKate2-FKBP-AP21967 for 1 min and washed with PBS. Transferrin-488 was added to the cells. VAMP2 and transferrin were observed to be internalized by endocytosis simultaneously and traffic together in the same vesicles (Fig. S1 and Supplementary movie), and they colocalized well at 30 min after labeling (Fig. S1B).

The use of purified mKate2-FKBP allowed most of the VAMP2-TagBFP-FRB (T2098L) on the cell surfaces to be labeled within tens of seconds (t_1/2_ of 13 s) with very high specificity. We demonstrated that constitutive VAMP endocytosis involves the same pathway as stimulated recycling in a clathrin-dependent pathway.

In summary, the LAPREP method we developed for the specific labeling of cell surface proteins offers several advantages over antibody-based labeling methods, namely the greater specificity and much smaller size of FKBP-fused fluorescent proteins compared with antibodies. This method also has several advantages over currently employed chemical-based labeling techniques, such as SNAP-tag and CLIP-tag. First, LAPREP has a high specificity for the labeling of surface proteins, even at high concentrations of FKBP-fused fluorescent proteins, whereas chemical dye-based labeling methods exhibit increased nonspecific binding at increased dye concentrations. Second, the low background fluorescence of the unreacted tag allows this method to be used for dynamic labeling without a washing step (Fig. 1). Third, the labeling rate is very fast, which allows high labeling efficiency and is useful for labeling proteins that cycle very rapidly between the intracellular compartments and the PM. Finally, the method is flexible and easy to use for the fluorescent labeling of multiple proteins or for pulse-chase labeling. The high specificity and affinity of FKBP-FP-AP21967 for FRB (T2098L) make it easy to wash away unbound FKBP-FP within several seconds and to exchange one fluorescent protein to another. The diversity and various properties of FPs will make this method extremely useful for pulse-chase and/or multicolor labeling. However, every coin has its two sides. LAPREP has a high specificity for labeling surface proteins, but it is different from permeable chemical dyes. LAPREP cannot be used to label the intracellular proteins because the purified FP-conjugated FKBP cannot cross membrane, so it has the limit for labeling cytoplasmic proteins in live cells.

## Footnotes

We thank ARIAD Pharmaceuticals, Inc. for providing AP21967 and the FKBP and FRB cDNAs. This work was supported by grants from the National Basic Research Program (973 Program) (Nos. 2010CB833701 and 2013CB910103), the National Natural Science Foundation of China (Grant Nos. 31130065, 31270910, 31370851, 31170818, and 31300612) and the CAS Project (KSCX2-EW-Q-11 and KSCX1-1W-J-3).

Xi Zhang, Yongqiang Deng, Hao Chang, Chen Ji, Mingshu Zhang, Jianxin Peng, Tao Xu, and Pingying Xu declare that they have no conflict of interest.

This article does not contain any studies with human or animal subjects performed by the any of the authors.

## Electronic supplementary material

Below is the link to the electronic supplementary material.

Supplementary material 1 (PDF 304 kb)

## References

[CR1] Banaszynski LA, Chen L-C, Maynard-Smith LA, Ooi AGL, Wandless TJ (2006). A rapid, reversible, and tunable method to regulate protein function in living cells using synthetic small molecules. Cell.

[CR2] Chen I, Howarth M, Lin W, Ting AY (2005). Site-specific labeling of cell surface proteins with biophysical probes using biotin ligase. Nat Methods.

[CR3] Dittman J, Ryan TA (2009). Molecular circuitry of endocytosis at nerve terminals. Annu Rev Cell Dev Biol.

[CR4] Eliasson L, Abdulkader F, Braun M, Galvanovskis J, Hoppa MB, Rorsman P (2008). Novel aspects of the molecular mechanisms controlling insulin secretion. J Physiol.

[CR5] George N, Pick H, Vogel H, Johnsson N, Johnsson K (2004). Specific labeling of cell surface proteins with chemically diverse compounds. J Am Chem Soc.

[CR6] Grote E, Kelly RB (1996). Endocytosis of VAMP is facilitated by a synaptic vesicle targeting signal. J Cell Biol.

[CR7] Harel A, Wu F, Mattson MP, Morris CM, Yao PJ (2008). Evidence for CALM in directing VAMP2 trafficking. Traffic.

[CR8] Hoffmann C, Gaietta G, Zurn A, Adams SR, Terrillon S, Ellisman MH, Tsien RY, Lohse MJ (2010). Fluorescent labeling of tetracysteine-tagged proteins in intact cells. Nat Protoc.

[CR9] Keppler A, Gendreizig S, Gronemeyer T, Pick H, Vogel H, Johnsson K (2003). A general method for the covalent labeling of fusion proteins with small molecules in vivo. Nat Biotech.

[CR10] Los GV, Encell LP, McDougall MG, Hartzell DD, Karassina N, Zimprich C, Wood MG, Learish R, Ohana RF, Urh M (2008). HaloTag: a novel protein labeling technology for cell imaging and protein analysis. ACS Chem Biol.

[CR11] Miller SE, Sahlender DA, Graham SC, Höning S, Robinson MS, Peden AA, Owen DJ (2011). The molecular basis for the endocytosis of small R-SNAREs by the clathrin adaptor CALM. Cell.

[CR12] Regazzi R, Wollheim C, Lang J, Theler J, Rossetto O, Montecucco C, Sadoul K, Weller U, Palmer M, Thorens B (1995). VAMP-2 and cellubrevin are expressed in pancreatic beta-cells and are essential for Ca(2+)-but not for GTP gamma S-induced insulin secretion. EMBO J.

[CR13] Sudhof TC (2004). The synaptic vesicle cycle. Annu Rev Neurosci.

[CR14] Sun X, Zhang A, Baker B, Sun L, Howard A, Buswell J, Maurel D, Masharina A, Johnsson K, Noren CJ (2011). Development of SNAP-tag fluorogenic probes for wash-free fluorescence imaging. ChemBioChem.

[CR15] Uttamapinant C, White KA, Baruah H, Thompson S, Fernández-Suárez M, Puthenveetil S, Ting AY (2010). A fluorophore ligase for site-specific protein labeling inside living cells. Proc Natl Academy Sci.

