# Supplementary material for: The specific and rapid labeling of cell surface proteins with recombinant FKBP-fused fluorescent proteins
Source: Protein Cell. 2014 Aug 12;5(10):800–3. doi: 10.1007/s13238-014-0090-8 (PMC4180455; doi:10.1007/s13238-014-0090-8)
Supplement: Supplementary file 1 — Supplementary material 1 (PDF 304 kb) [file 13238_2014_90_MOESM1_ESM.pdf]

Supporting Information

The specific and rapid labeling of cell surface proteins with recombinant FKBP-fused fluorescent proteins

Xi Zhang[1],Yongqiang Deng[2], Hao Chang[3], Chen Ji[2], Mingshu Zhang[4], Jianxin Peng[1], Tao Xu[2], Pingyong Xu[4]

[1] Institute of Entomology, School of Life Sciences, Central China Normal University, Wuhan, 430079, China

[2]National Key Laboratory of Biomacromolecules, Institute of Biophysics, Chinese Academy of Sciences, Beijing 100101, China

[3] School of Life Sciences, University of Science and Technology of China, Hefei, Anhui, 230026, China

[4] Laboratory of Interdisciplinary Research, Institute of Biophysics, Chinese Academy of Sciences, Beijing 100101, China

Correspondence: Tao Xu, Tel/Fax: +86-10-64888524, E-mail: xutao@ibp.ac.cn; Pingyong Xu, Tel/Fax: +86-10-64888808, E-mail: pyxu@ibp.ac.cn; Jianxin Peng, Tel/Fax: +86-027-64862431, E-mail: jxpeng@mail.ccnu.edu.cn

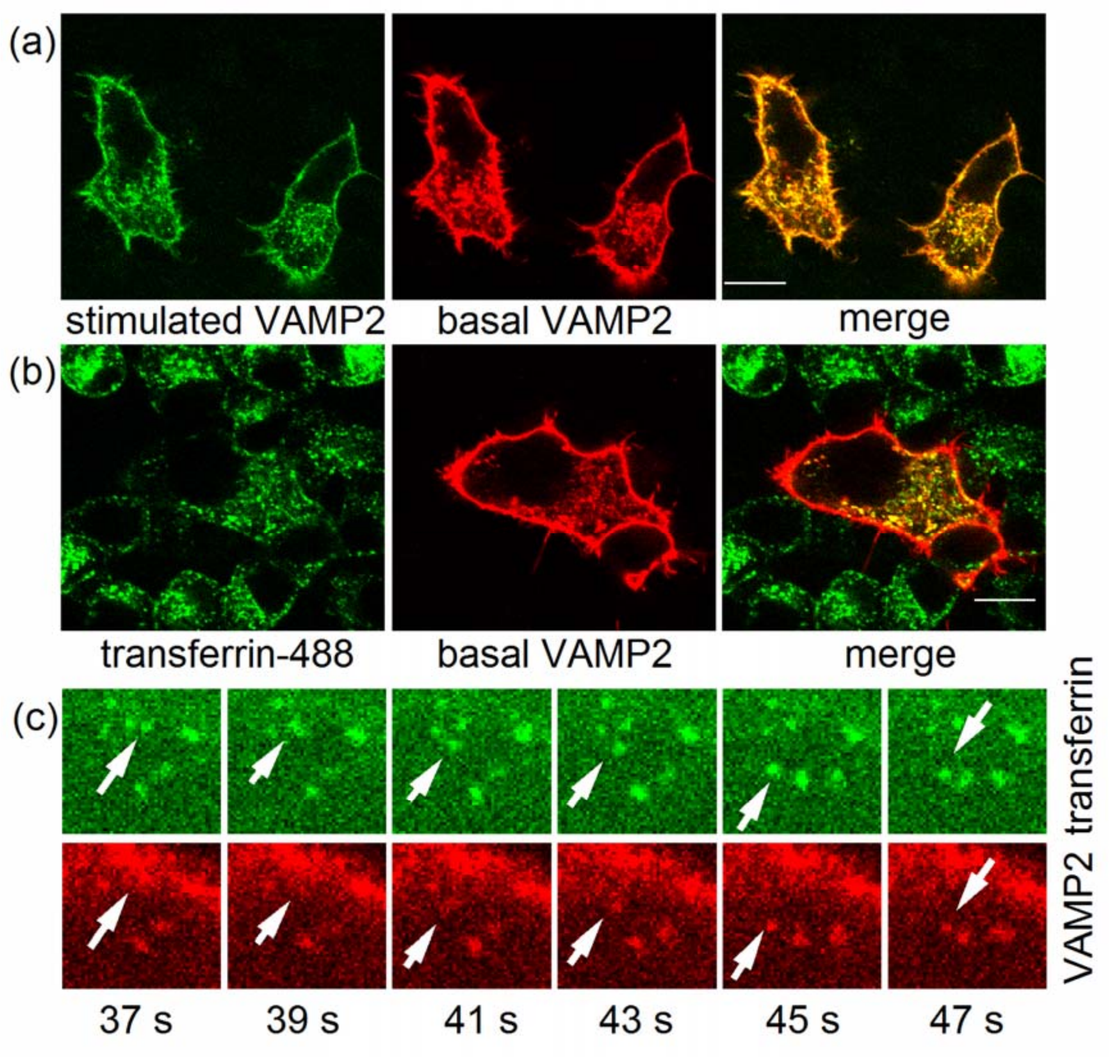

Figure S1 Application FKBP-AP21967 to VAMP2 endocytosis. (a) Basal VAMP2 representing constitutive insulin secretion was first labeled with mKate2-FKBP-AP21967. Stimulated VAMP2 representing regulated insulin secretion was labeled with GFP-FKBP-AP21967. They colocalized very well after entering the cells, indicating both VAMP2 endocytosis shared the same pathway. (b) PM basal VAMP2 endocytosis underwent clathrin-dependent pathway as transferrin. (c) VAMP2 was internalized and trafficking simultaneously with transferrin in the same vesicle as indicated by the arrows.

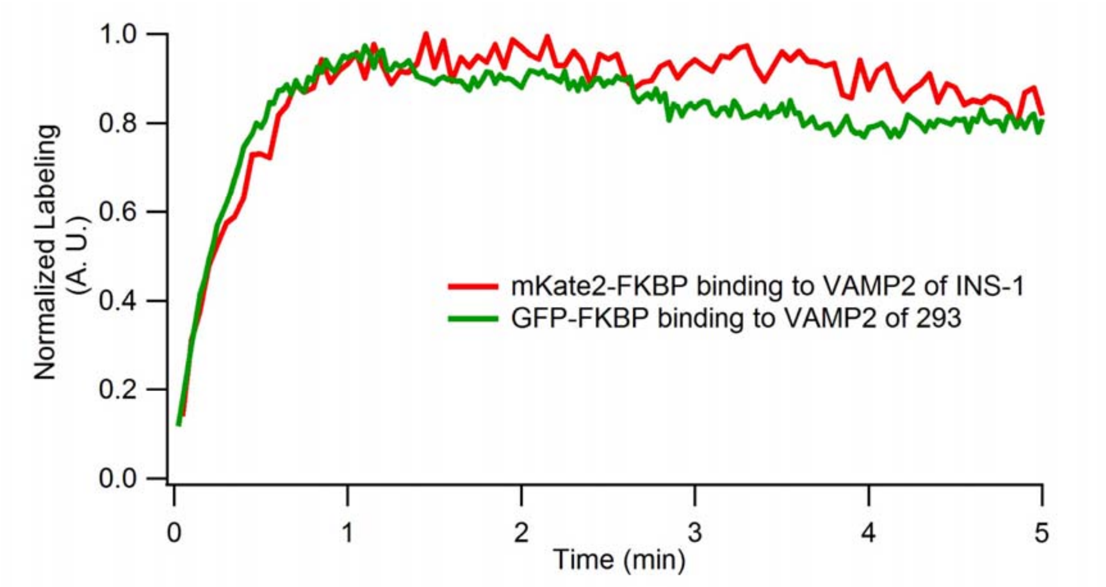

Figure S2 mKate2-FKBP and EGFP-FKBP binding to VAMP2-FRB (T2098L) on the PM are quite fast.

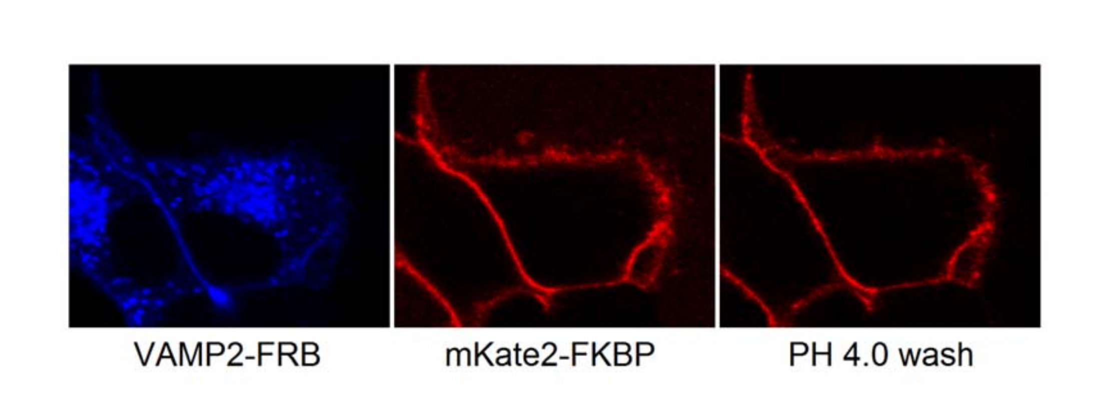

Figure S3 The FKBP-AP21967-FRB (T2098L) ternary complex are very stable even washed with high salt, low PH buffer (0.5% acetic acid, 0.5 M NaCl, pH 4.0).

Movie S1 mKate2-FKBP- AP21967 labeling VAMP2-FRB and transferrin-488 endocytosis in the same vesicles in the live cells.

Materials and Methods

General Methods

The rapalog AP21967 and cDNA for FKBP and FRB (T2098L) was a kindly gift from ARIAD pharmaceuticals, and ascomycin was obtained from Sigma-Aldrich. Pfu polymerase, restriction enzymes, T4 DNA ligase and SNAP-Surface 488 were obtained from NEB. The primers for PCR and Alexa Fluor 488-conjugated transferrin were purchased from Invitrogen. Unless otherwise noted, all of the remaining chemical reagents were purchased from Sigma.

The concentrations of the recombinant proteins were measured on a Biodropsis UV spectrophotometer (Wuzhou Beijing Orient). The microscopy experiments were performed on an FV1000 confocal laser scanning microscope (Olympus Optical Co., Tokyo, Japan). The experiments were conducted using a 60× oil objective. The images were processed using the FV1000 software package FluoView and Image J (NIH).

The cloning, expression and purification of pRSETa-mKate2-FKBP

The plasmid pRSETa-mKate2-FKBP was constructed as follows: The fluorophore mKate2 was amplified by PCR with primers mKate2-up (AAA GGA TCC ATG GTG AGC GAG CTG ATT AAG) and mKate2-rev (CTG CAG GGT GCC GCC AGA GCC TCC TCT GTG CCC CAG TTT GCT AGG). FKBP was amplified with the primers FKBP-up (GGA GGC TCT GGC GGC ACC CTG CAG GGA GTG CAG GTG GAA ACC ATC TC) and FKBP-rev (AAA GAA TTC TTA ATG CAT TTC CAG TTT TAG AAG CTC CAC). The mKate2 and FKBP PCR products were mixed,

and the primers mKate2-up and FKBP-rev were used to perform PCR to generate the mKate2-FKBP chimera. The mKate2-FKBP fragment was digested with *Bam*H1 and *Eco*R1 and inserted into pRSETa. This plasmid was transformed into *E. coli* TOP10 competent cells and grown overnight on LB agar plates with ampicillin. Colonies from the agar plates were transferred into 5 ml LB with ampicillin, grown overnight at 37°C and then sequenced (Invitrogen) to confirm that the construct was correct.

The plasmid DNA pRSETa-mKate2-FKBP was purified with the TIAN prep Mini Plasmid Kit (Tiangen, Beijing) and transformed into *E. coli* strain BL21 (DE3) cells. The cells were grown on agar plates containing ampicillin, picked the single clone and transferred into 5 ml LB with ampicillin, and grown overnight at 37°C. Subsequently, 500 mL of LB medium was inoculated with the 5 ml overnight cultures and grown at 37 °C at 200 rpm until the optical density (OD) at 600 nm reached 0.6 ~ 0.7. IPTG (1 mM) was added for protein induction, and the culture was further grown at 20 °C for 8 h. The cells were collected by centrifugation at 8000 *g* for 5 min. The pellet was then resuspended in 30 mL of lysis buffer (50 mM Tris, 500 mM NaCl and 5 mM imidazole, pH 8.0) and lysed by sonication on ice. The mixture was centrifuged at 18,000 *g* for 30 min, yielding a clear lysate containing the target protein mKate2-FKBP. The lysate was loaded in a Ni-NTA column (Qiagen) that was previously balanced three times with lysis buffer. The loaded column was then washed three times with wash buffer (50 mM Tris, 500 mM NaCl and 50 mM imidazole, pH 8.0) to remove the nonspecific binding proteins. Elution buffer (50 mM Tris, 500 mM NaCl and 500 mM imidazole, pH 8.0) was added to the column to elute mKate2-FKBP, and the flow-through was collected.

The fraction containing mKate2-FKBP was further purified in PBS by gel filtration chromatography using a Superdex 200 column (GE Healthcare). The purified mKate2-FKBP protein was then concentrated in a centrifugal concentrator with a molecular weight cut-off of 10 kDa (Millipore) and stored at -80°C. The concentration of mKate2-FKBP was determined by measuring the absorbance at 280 nm with a Biodropsis UV spectrophotometer (Wuzhou Beijing Oriental).

The cloning, expression and purification of pRSETa-EGFP-FKBP

The EGFP coding fragment was amplified by PCR from N1-EGFP using the primers EGFP-up (CCG GGA TCC ATG GTG AGC AAG GGC GAG GAG) and EGFP-rev (ATT CTG CAG GGT GCC GCC AGA GCC TCC CTT GTA CAG CTC GTC CAT GCC). This fragment was digested with *Bam*H1 and *Pst*1 and replaced mKate2 in pRSETa-mKate2-FKBP to generate pRSETa-EGFP-FKBP. The plasmid was transformed into *E. coli* TOP10 competent cells, and the correct construction was confirmed by direct sequencing. pRSETa-EGFP-FKBP plasmid DNA was purified with the TIAN prep Mini Plasmid Kit and transformed into *E. coli* strain BL21 (DE3) cells.

Liquid cultures of BL21 (DE3) *E. coli* cells containing pRSETa-EGFP-FKBP were grown at 37°C until their OD_{600} was approximately 0.7. The expression of 6xHis-EGFP-FKBP was induced by adding IPTG to a final concentration of 1 mM. After incubation at 200 rpm and 20°C for 8 hours, the culture was collected by 5 min of centrifugation at 8000 *g* at 4°C. The EGFP-FKBP was purified as mentioned above.

The concentration of EGFP-FKBP was determined by measuring the absorbance at 280 nm with a Biodropsis UV spectrophotometer (Wuzhou Beijing Oriental).

The cloning of VAMP2-TagBFP-FRB (T2098L) and VAMP2-TagBFP-SNAP

VAMP2 was amplified by PCR using the primers VAMP2-up (AAA GGA TCC CCA CCA TGT CGG CTA CCG CTG CCA CCG) and VAMP2-rev (TCC ACC GGT CCC GCC GCT TCC GCC GCT AGT GCT GAA GTA AAC GAT GAT G). TagBFP was amplified using the primers TagBFP-up (AGC GGC GGA AGC GGC GGG ACC GGT GGA AGC GAG CTG ATT AAG GAG AAC) and TagBFP-rev (GGT TCC TCC GCT CCC TCC ACG CGT GTG CCC CAG TTT GCT AGG GAG GTC GC). FRB was amplified with the primers FRB-up (ACG CGT GGA GGG AGC GGA GGA ACC ATC CTC TGG CAT GAG ATG TGG C) and FRB-rev (ATA GCG GCC GCT TAT GCG TAG TCT GGT ACG TCG TAC GG). The three fragments were linked together by PCR with the primers VAMP2-up and FRB-rev. The N1-EGFP vector was digested with *Bam*H1 and *Not*1 to replace EGFP with the VAMP2-TagBFP-FRB fragment. The plasmid VAMP2-TagBFP-FRB (T2098L) was sequenced to confirm correct construction. The plasmid DNA VAMP2-TagBFP-FRB (T2098L) from 100 ml *E. coli* cultures was purified using the Wizard plus Midiprep DNA Purification System (Promega).

VAMP2-TagBFP-SNAP was constructed as follows. VAMP2-TagBFP was amplified from the plasmid VAMP2-TagBFP-FRB using the primers VAMP2-up2 (AAA CTC GAG CCA CCA TGT CGG CTA CCG CTG CCA CCG) and TagBFP-rev2 (AAA GGA TCC GGA AGC TTG TGC CCC AGT TTG CTA GG

GAG). The PCR product was digested with *Xho*1 and *Bam*H1 and inserted in-frame into N1-SNAPtag.

Cell surface protein labeling

HEK293 cells were cultured in Dulbecco's modified Eagle's medium (DMEM) supplemented with 10% fetal bovine serum. Cells were transfected with VAMP2-TagBFP-FRB (T2098L) using Lipofectamine^{TM} 2000 (Invitrogen) according to the manufacturer's instructions. After 24 hours, the cells were digested with trypsin and plated on coverslips that had been coated with poly-lysine. After another 12-24 hours, the transfected cells were incubated with 1 μM mKate2-FKBP mixed with AP21967 (1 μM) for 1 minute and then washed three times with PBS. The cells were observed with a confocal laser scanning microscope (FV1000, Olympus) using a 60 × oil objective.

AP21967-dependent protein labeling

HEK293 cells were transfected and treated as described above. The cells that were transfected with VAMP2-TagBFP-FRB (T2098L) were subjected to fluorescence microscopy (FV1000, Olympus), and the cells with a high expression level of VAMP2-TagBFP-FRB (T2098L) on the PM were selected by monitoring TagBFP with a 405 nm laser. The cells were first incubated with 1 μM mKate2-FKBP without AP21967 for 5 min at room temperature. Images were obtained bying 559 nm illumination, then cells were washed for three times with PBS and incubated with 1 μM mKate2-FKBP mixed with AP21967 (1 μM) for 1 min, and images were acquired without further washing.

The inhibition of labeling with ascomycin

HEK293 cells were transfected and treated as above. Cells transiently expressing VAMP2-TagBFP-FRB (T2098L) were incubated in PBS containing 1 μM mKate2-FKBP mixed with 1 μM AP21967 and 5 μM ascomycin. The cells were subjected to fluorescence microscopy after 10 min of labeling without washing with PBS. The same cells were then washed three times with PBS and incubated with1 μM mKate2-FKBP mixed with AP21967 (1 μM) for 1 min, and images were again acquired without a preceding washing step.

The kinetics of mKate2-FKBP-AP21967 labeling

HEK293 cells transiently expressing VAMP2-TagBFP-FRB (T2098L) were subjected to FV1000 fluorescence microscope, and cells with similar levels of FRB protein expression on the cell surface were selected by monitoring TagBFP with a 405 nm laser. The same amount of mKate2-FKBP (1 μM) mixed with different concentrations of AP21967 (50 nM, 100 nM, 250 nM and 1 μM) was added to the selected cells. Images were acquired in real time (1 image/3 s) during the labeling reaction. The laser intensity and acquisition parameters were adjusted to avoid saturating the image or damaging the cells. To compare the intensity of the bound fluorescent protein, all of the acquisition parameters were held constant during the process of labeling with different concentrations of the AP21967-FKBP complex. The fluorescence intensity of the background and the cell surface were measured in Image J. The specific mKate2 fluorescent labeling signals were calculated by subtracting the background fluorescence from the cell surface fluorescence. The ultimate fluorescence

intensity of each concentration of AP21967 at a specific timepoint was averaged from at least 5 cells. The FKBP-FRB labeling kinetics was analyzed by Igor Pro 6.11. The $t_{½}$ values were calculated based on curve-fitting analyses.

The kinetics of SNAP-Surface 488 labeling

HEK293 cells transfected with VAMP2-TagBFP-SNAP were observed with an FV1000 fluorescence microscope, and cells with similar expression levels of SNAP tag proteins and VAMP2-TagBFP-FRB were selected by monitoring TagBFP with a 405 nm laser. The cells were incubated with 1 μM SNAP-Surface 488. Images were acquired and processed as described above.

VAMP2 translocation time-course experiments

INS-1 cells were grown at 37°C in 5% CO_2 in RPMI-1640 (Gibco) with 10% fetal bovine serum, 1 mM sodium pyruvate and 50 mM 2-mercaptoethanol. The cells were transfected with VAMP2-TagBFP-FRB (T2098L) using Lipofectamine^{TM} 2000 (Invitrogen). The transfected cells were subjected to fluorescence microscopy, and cells with similar FRB protein expression levels were selected by monitoring TagBFP with a 405 nm laser. The selected cells were incubated in Krebs-Ringer bicarbonate buffer (KRBB) containing mKate2-FKBP-AP21967 complex (1 μM mKate2-FKBP mixed with 1 μM AP21967) for 5 min to block the preexisting VAMP2 on the PM. The cells were then washed three times with PBS to remove the unbound mKate2-FKBP. The EGFP-FKBP-AP21967 complex was added to the cell surface, either in basal KRBB to monitor the constitutive insulin secretion and VAMP2 translocation to the PM or in KRBB containing 30 mM glucose and 30 mM KCl to monitor the regulated VAMP2

translocation. Images were acquired at 30 s and at 1, 2, 5, 8, 11, 14, 17 and 20 min. The labeling fluorescence intensity was measured as described above. The EGFP labeling represents the newly arriving VAMP2. The KRBB solution contained 129 mM NaCl, 4.7 mM KCl, 1.2 mM KH_2PO_4, 5 mM $NaHCO_3$, 10 mM HEPES, 3 mM glucose, 2.5 mM $CaCl_2$, 1.2 mM $MgCl_2$ and 0.1% BSA (pH 7.2).

VAMP2 endocytosis

INS-1 cells transfected with VAMP2-TagBFP-FRB (T2098L) were manipulated as described for VAMP2 translocation. After the cells were labeled with EGFP-FKBP for 30 min and washed three times with PBS, they were incubated in PBS for another 15 min to allow the endocytosis of the two fractions of the VAMP2 molecules. The cells were subjected to FV1000 fluorescence microscopy with 405 nm, 488 nm and 559 nm lasers used for image acquisition.

INS-1 cells were transfected with VAMP2-TagBFP-FRB (T2098L). The transfected cells were starved for 2 hours in serum-free RPMI-1640 in the presence of 1 mg/ml BSA at 37°C. The cells were first incubated in PBS containing 1 μM mKate2-FKBP mixed with 1 μM AP21967 for 1 min to label the VAMP2 molecules. The cells were then washed three times with PBS and then incubated with 10 μg/ml Alexa Fluor® 488-conjugated transferrin (Invitrogen) for 20 min at 37°Cmin. The cells were then subjected to fluorescence microscopy after washes with PBS three times.

Hela cells expressing VAMP2-TagBFP-FRB (T2098L) were starved for 2 hours in serum-free DMEM in the presence of 2 mg/ml BSA at 37°C. The cells with the highly expression level of TagBFP-FRB were selected and subjected to confocal

microscope spin-disk. The cells were first incubated in PBS containing 1 μM mKate2-FKBP mixed with 1 μM AP21967 for 1 min to label the VAMP2 molecules. The cells were then washed three times with PBS and incubated with 30 μg/ml Alexa Fluor® 488 Conjugate transferrin (Invitrogen). The images were acquired in real time with lasers at 488 nm and 561 nm on a Zeiss Observer Z1 microscope (Carl Zeiss) with a 60 × oil objective. The exposure time is 200 ms for 488 nm and 300 ms for 561 nm. The gain of EM-CCD was set at 200.
